# A Direct Phenotypic Comparison of siRNA Pools and Multiple Individual Duplexes in a Functional Assay

**DOI:** 10.1371/journal.pone.0008471

**Published:** 2009-12-29

**Authors:** Brendon D. Parsons, Anja Schindler, David H. Evans, Edan Foley

**Affiliations:** Alberta Institute for Viral Immunology, Department of Medical Microbiology and Immunology, University of Alberta, Edmonton, Alberta, Canada; Oregon State University, United States of America

## Abstract

**Background:**

RNAi is a prominent tool for the identification of novel regulatory elements within complex cellular pathways. In invertebrates, RNAi is a relatively straightforward process, where large double-stranded RNA molecules initiate sequence-specific transcript destruction in target cells. In contrast, RNAi in mammalian cell culture assays requires the delivery of short interfering RNA duplexes to target cells. Due to concerns over off-target phenotypes and extreme variability in duplex efficiency, investigators typically deliver and analyze multiple duplexes per target. Currently, duplexes are delivered and analyzed either individually or as a pool of several independent duplexes. A choice between experiments based on siRNA pools or multiple individual duplexes has considerable implications for throughput, reagent requirements and data analysis in genome-wide surveys, yet there are relatively few data that directly compare the efficiency of the two approaches.

**Methodology/Principal Findings:**

To address this critical issue, we conducted a direct comparison of siRNA pools and multiple single siRNAs that target all human phosphatases in a robust functional assay. We determined the frequency with which both approaches uncover loss-of-function phenotypes and compared the phenotypic severity for siRNA pools and the constituent individual duplexes.

**Conclusions/Significance:**

Our survey indicates that screens with siRNA pools have several significant advantages over identical screens with the corresponding individual siRNA duplexes. Of note, we frequently observed greater phenotypic penetrance for siRNA pools than for the parental individual duplexes. Thus, our data indicate that experiments with siRNA pools have a greater likelihood of generating loss-of-function phenotypes than individual siRNA duplexes.

## Introduction

Reverse genetic studies are a dominant force in the functional characterization of eukaryotic gene products in many pertinent model systems. Unfortunately, such studies tend to be laborious, costly and time-consuming in complex eukaryotic models. Thus, whereas targeted loss-of-function studies are commonplace in comparatively straightforward organisms such as yeast, *in vivo* mutational analyses present considerable challenges in subjects of a more immediate biomedical relevance such as mice. The recent advent of the “RNA universe” fundamentally altered perspectives for reverse genetic studies in mammalian systems. Specifically, RNA interference (RNAi) bears the real promise of rapid and directed genome-wide loss-of-function studies in a broad range of cell culture models [1]. The principles of genome-scale RNAi screens were first established in *C. elegans* and *Drosophila melanogaster* and were later applied to mammalian systems [2], [3], [4]. Fortuitous parallel developments of automated systems such as liquid handling devices and high-throughput plate readers place genome-wide studies of direct biomedical relevance within immediate reach. For example, RNAi screens in mammalian cell culture assays successfully probed topics as diverse as embryonic stem cell self-renewal, West Nile virus infection and various aspects of cancer progression [5], [6], [7], [8], [9]. Looking forward, progress in assay miniaturization [10] and the establishment of high-content systems makes it clear that more sophisticated assays are yet to appear in the RNAi toolbox.

Despite these astounding developments, it is clear that RNAi is not the ultimate panacea for the contemporary cell biologist. A particular vexing issue that has not been fully addressed in siRNA screens is the question of knock-down efficiency. In this context, a major decision in every siRNA experiment is the amount of siRNA duplexes to deliver for each gene target. Current conventions suggest that a minimum of three siRNA duplexes that target non-overlapping sections of the same gene product should be employed. There are two theoretical advantages to such an approach. First, as many siRNA duplexes fail to significantly deplete their intended target, increased numbers of duplexes should increase the likelihood of generating the true loss-of-function phenotype for a particular gene. Furthermore, the ability to probe a given target with multiple siRNA duplexes should decrease the impact of off-target effects on hit identification, if one sets a threshold that a minimum of two non-overlapping siRNA duplexes should yield an overlapping phenotype [11]. Based on these considerations, the standard practice for many siRNA experiments is to test a minimum of three non-overlapping duplexes and exclude all gene products that were not affected by a minimum of two siRNA duplexes.

There are currently two approaches to test multiple siRNA duplexes in genome-scale screens. In one case, each duplex is tested individually. Whereas this approach requires considerable commitments in terms of resources, it permits stratification of hits into confidence groups based on the number of individual siRNA duplexes that uncover a particular phenotype. The alternative approach is to test all siRNA duplexes that target a given gene product as a single pool. The obvious advantage to such an approach is that it maximizes resources and increases throughput. However, siRNA pool screens do not provide insights into the number of individual siRNAs that contribute to a given phenotype and the onus is on the investigator to test multiple single siRNA duplexes in secondary assays specifically designed to probe false positive rates. Both approaches are routinely applied in genome-scale screens, yet the relative merits of siRNA pools and single siRNA duplexes have not been tested in a systematic phenotypic assay. Given the clear ramifications such considerations have for screen execution, we consider this a critical issue.

Here, we present the results of a direct comparison of the behavior of pooled and single siRNA duplexes in an unbiased functional screen of the entire set of human phosphatases. Our data indicate that the apparent hit frequency is higher in screens performed with siRNA pools and that phenotypes from siRNA pools are often more pronounced than phenotypes for the corresponding individual siRNA duplexes. These observations combined with the advantages represented by siRNA pools in terms of throughput and reagent consumption, lead us to propose that siRNA pools are a superior tool for genome-scale siRNA screens.

## Results

### A Quantitative siRNA-Based Assay for Modifiers of TNF-Dependent Cell Death

We developed a quantitative plate-based assay to identify siRNA duplexes that modify cellular responses to the Tumor Necrosis Factor-α (TNF) cytokine. Engagement of the Tumor Necrosis Factor Receptor (TNFR) by homotrimeric TNF ligands drives the activation of JNK, NF-κB and caspase signal transduction cassettes. NF-κB family members promote the transcription of “pro-survival” gene products that contribute to inflammatory responses, induce cellular proliferation and differentiation and actively block caspase-mediated pro-apoptotic events. In the absence of NF-κB-responsive gene products, TNF signal transduction results in apoptotic cell death through the caspase cascade.

**Figure 1:**
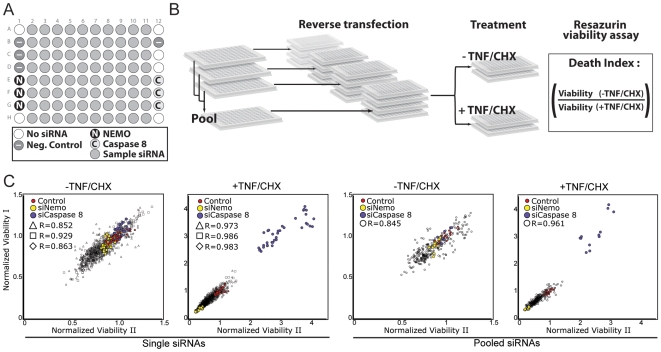
A plate-based siRNA screen for modifiers of TNF-induced cell death. (A) Phosphatase siRNA plate layout for each plate of the screen. Positive control siRNAs target caspase-8 or NEMO and negative control siRNAs do not silence any cellular target. (B) Schematic representation of screen preparation and workflow. The ratio of the viability score from untreated cells to the viability of treated cells represents the TNF-induced death (death index). (C) Viability measurements for replicate assays. The viability scores for each plate are plotted against the viability scores of the corresponding replicate. Plots represent the comparison of replicate plates incubated with either single or pooled siRNAs and treated with TNF/Cycloheximide (CHX) as indicated.

We used a defined set of siRNA duplexes to probe the entire annotated collection of human phosphatases (265 individual genes) for modifiers of TNF-dependent cell death. The layout of each siRNA master plate is shown in Figure 1A. Each plate contained 80 experimental siRNA duplexes that target defined phosphatases. In addition, each master plate contained multiple control wells, such as no siRNA duplexes, a non-silencing control siRNA duplex and positive control siRNA duplexes that target NEMO or caspase-8. We chose NEMO and caspase-8 as controls, as they are essential for the activation of NF-κB or the induction of apoptotic events, respectively. We prepared four master plates for each target gene: three plates contained unique non-overlapping siRNA duplexes and the fourth plate contained a pool of the three individual duplexes. Each master plate was prepared in such a way that all plates contained equimolar concentrations of total siRNA.

We then screened each plate for modifiers of TNF-dependent cell death. The screen layout is described in Figure 1B. We treated two replicate plates with TNF and non-lethal doses of the translation inhibitor cycloheximide. The combined regime attenuates the expression of NF-κB responsive anti-apoptotic factors and is a widespread method for the induction of TNF-responsive cell death in a number of different cell lines. We quantified cell viability in each well under the respective culture conditions in a resazurin cell viability assay and normalized each viability score to the mean of the non-silencing control siRNA on the same plate. Normalization allowed us to perform plate-to-plate comparisons and develop a comparable “death index” to assess the level of TNF-induced death for each siRNA under scrutiny.

Analysis of replicate measurements confirmed that the overwhelming majority of the cell viability measurements was reproducible (Figure 1C). Control caspase-8 siRNAs reliably blocked TNF/cycloheximide-dependent cell death, while NEMO siRNAs reproducibly increased TNF/cycloheximide-dependent death. Both phenotypes are consistent with the respective roles of caspase-8 and NEMO in the induction or inhibition of TNF-dependent apoptosis. Statistical evaluation of screen data confirmed that the overall screen quality was high [12]. In summary, we are confident that the assay represents a valid springboard for the identification of siRNA duplexes that modify TNF-dependent cell death.

### Statistical Evaluation of Screen Data

**Figure 2:**
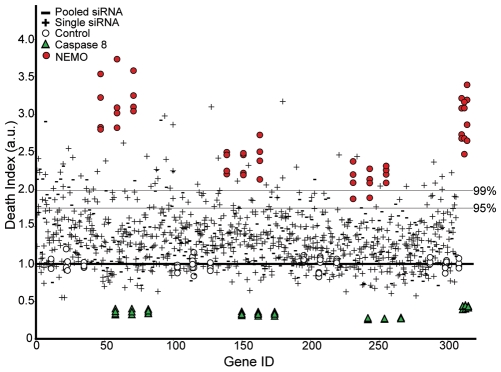
Two siRNA screening approaches identify modifiers of TNF-induced cell death. Target genes are shown on the x-axis in the order screened with each corresponding experimental viability score normalized and plotted as a distribution in the y-axis around the mean viability score of the non-silencing siRNA plate control, which was set to a death index of one. The 95% and 99% confidence intervals shown represent the probability threshold of TNF modifiers amongst the single siRNAs screened. The death indices of control siRNAs from each master plate are shown.

We then plotted the death index for each siRNA from the primary screen (Figure 2). The corresponding raw viability measurements are available in Table S1. For statistical purposes, we defined a modifier siRNA as an siRNA duplex that produced a death index greater than 1.96 standard deviations above the median non-silencing control siRNA. These criteria define the 95 percent confidence interval for true modifier siRNAs and are routinely applied for “hit” identification in high-throughput RNAi screens. As we consider assaying a pool of three siRNA duplexes a distinct experimental approach to an assay that probes each siRNA duplex separately, we identified modifier siRNA pools and modifier single siRNA duplexes separately. Current standards for experiments with single siRNA duplexes dictate that a minimum of two non-overlapping siRNAs are required to produce overlapping phenotypes before the target gene product should be considered a valid modifier. Based on these critieria, we defined two separate categories of modifier single siRNAs: a high-confidence set, where all three siRNAs fell within the 95% confidence interval; and a medium-confidence set, where two of three siRNAs fell within the 95% confidence interval.

### A Comparison of Hits from the Single and Pooled siRNA Duplexes

**Figure 3:**
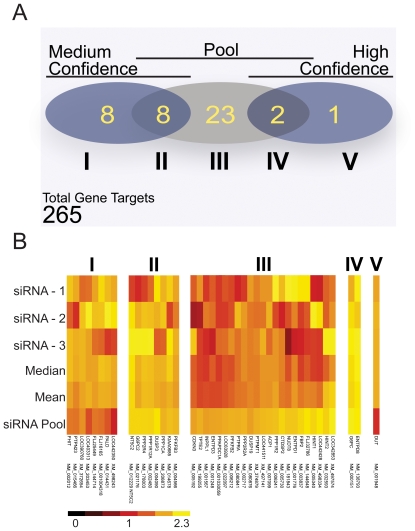
Relationship between putative hits from siRNA pools or single siRNA duplexes. (A) The number and relation of gene target hits from single and pooled siRNA screens. The Venn diagram shows high and medium confidence hit groups from the single siRNA screen and indicates the degree to which each group overlaps with the siRNA pool hits. (B) Hierarchical cluster analysis of pool screen hits and medium to high confidence interval single screen hits. The death index of siRNA targets are clustered on a color scale ranging from the highest death index value, depicted as yellow, to the lowest death index value, depicted as black, and centered around the non-silencing control assigned a death index of one, depicted as red. Group I represents medium confidence single siRNA hits. Group II represents all putative hits identified in the pool and medium confidence single siRNA screen. Group III represents all putative hits identified in the pool screen only. Group IV represents all putative hits identified in the pool and high-confidence single siRNA screen. Group V represents high confidence single siRNA hits.

We then compared modifier gene products identified through pooled siRNAs and single siRNA assays. From these comparisons, we distinguished three broad classes of putative hits: “dual” hits, where the pool and a minimum of two single siRNA duplexes identified the target as a modifier; “pool only” hits, where only the pooled siRNA identified the target as a modifier; and “single only” hits, where a minimum of two single siRNA duplexes identify the target as a hit, but the pooled siRNA does not (Table 1). In total, we identified thirty three putative hits from the siRNA pools and nineteen putative hits from the individual siRNA duplexes, of which three were high-confidence modifiers and sixteen were medium-confidence modifiers (Figure 3A and B). Thus, while we detected a general overlap between both screens in terms of “hit” identification, our survey indicates that the frequency of hit identification is considerably higher for screens performed with siRNA pools.

**Table 1:** Putative screen hits.

Accession	GENE ID	GENE NAME	Hit Validity	Hit Confidence	Pool	siRNA 1	siRNA 2	siRNA 3
NM_000151	G6PC	glucose-6-phosphatase, catalytic	Dual Hit	High	2.9003	1.8459	2.1934	2.3147
NM_138793	ENTPD8	calcium activated nucleotidase 1			2.3062	2.3315	2.0295	2.4356
NM_004090	DUSP3	dual specificity phosphatase 3		Medium	2.2307	2.2403	1.8716	1.3754
NM_021176	G6PC2	glucose-6-phosphatase, catalytic, 2			2.1342	1.1284	1.8567	2.4528
NM_012229	NT5C2	5-nucleotidase, cytosolic II			2.1316	1.2446	2.2222	2.9737
NM_178003	PPP2R4	protein phosphatase 2A, regulatory subunit B			2.1239	1.2425	2.0035	2.6130
NM_002480	PPP1R12A	protein phosphatase 1, regulatorysubunit 12A			2.1128	1.4400	1.9571	2.4310
NM_014678	KIAA0685	KIAA0685			1.9730	2.0715	1.2485	2.2899
NM_004566	PFKFB3	6-phosphofructo-2-kinase/fructose-2,6-biphosphatase 3			1.9234	1.7908	1.5559	1.8546
NM_206873	PPP1CA	protein phosphatase 1, catalytic subunit, alpha isoform			1.8867	1.8826	2.0215	1.4203
XM_497574	LOC342853	–	Pool Only	Low	2.9180	1.5194	1.6771	3.0943
XM_498334	LOC442428	–			2.2651	0.9931	2.2196	1.1948
NM_144648	FLJ32786	FLJ32786			2.2541	2.2595	1.3660	1.1998
NM_003837	FBP2	fructose-1,6-bisphosphatase 2			2.1309	2.2476	1.2325	1.0972
NM_005730	CTDSP2	RNA pol. II c-terminal domain, polypeptide A small phosphatase 2			2.1289	1.9402	1.0854	1.5817
NM_005340	HINT1	histidine triad nucleotide binding protein 1			2.1233	1.0903	2.4192	1.2247
NM_032593	HINT2	histidine triad nucleotide binding protein 2			2.0986	1.4094	1.3917	3.1661
NM_001776	ENTPD1	ectonucleoside triphosphate diphosphohydrolase 1			2.0248	2.5402	1.5904	0.9978
NM_181843	NUDT8	nucleoside diphosphate linked moiety X-type motif 8			2.0241	2.3944	1.3122	0.7565
NM_006241	PPP1R2	protein phosphatase 1, regulatory subunit 2			1.9775	2.3725	1.2826	1.6937
NM_007099	ACP1	acid phosphatase 1, soluble			1.9499	1.6933	1.8857	1.4492
XM_497141	LOC441511	–			1.9292	1.6908	1.5069	1.8579
NM_080841	PTPRA	protein tyrosine phosphatase, receptor type, A			1.8600	1.1012	1.9511	1.4844
NM_005192	CDKN3	cyclin-dependent kinase inhibitor 3			1.7696	1.3822	0.8408	2.0323
NM_080876	DUSP19	dual specificity phosphatase 19			1.7569	1.7371	1.5304	1.5611
NM_002717	PPP2R2A	protein phosphatase 2, regulatory subunit B, alpha isoform		Zero	1.8898	1.2037	1.7246	1.6531
NM_006212	PFKFB2	6-phosphofructo-2-kinase/fructose-2,6-biphosphatase 2			1.8793	1.2788	1.3083	1.6599
XM_374879	PTPMT1	protein tyrosine phosphatase, mitochondrial 1			1.8792	1.5690	1.4851	1.6204
NM_199255	TPTE2	Transmemb. phosphoinositide 3-phosphatase & tensin homolog 2			1.8629	1.6875	0.8722	1.2343
NM_022097	LOC63928	LOC63928			1.8397	1.3315	1.2093	1.4714
NM_001030059	PPAPDC1A	phosphatidic acid phosphatase type 2 domain containing 1A			1.7969	1.2003	1.2035	1.4436
NM_001248	ENTPD3	ectonucleoside triphosphate diphosphohydrolase 3			1.7852	1.3722	1.4811	0.9802
NM_001567	INPPL1	inositol polyphosphate phosphatase-like 1			1.7479	1.1947	1.5363	1.1470
NM_001948	DUT	dUTP pyrophosphatase	Singles Only	High	1.1402	1.7850	1.9004	1.8668
NM_000507	LOC390760	–		Medium	1.6718	1.4416	1.9962	1.9126
NM_002012	FHIT	fragile histidine triad gene			1.5280	2.0044	1.3869	1.9565
NM_144714	FLJ25449	FLJ25449			1.5197	1.7870	1.9786	1.4684
NM_015466	PTPN23	protein tyrosine phosphatase, non-receptor type 23			1.4990	1.8296	1.1512	1.8577
NM_001004318	FLJ16165	FLJ16165			1.4731	2.1641	1.9933	1.4317
NM_014431	PALD	KIAA1274			1.3414	1.9367	2.4109	1.1976
NM_203453	LOC403313	LOC403313			1.2974	1.4860	2.3583	1.7807
XM_498243	LOC442350	–			1.0191	1.8247	2.2319	1.2570

The death index for each pool and single siRNA are shown in the final four columns. Putative hits are divided into groups according to the screens in which they were identified as modifiers. Putative modifiers are further subdivided into confidence intervals based on the number of single siRNAs that give a particular phenotype. In the pool only list, low confidence hits are genes for which a single siRNA gave a significant modifier phenotype, and zero confidence hits were not significantly modified by any single siRNA.

### Phenotypic Distinctions between Screens with siRNA Pools and Screens with Single siRNA Duplexes

**Figure 4:**
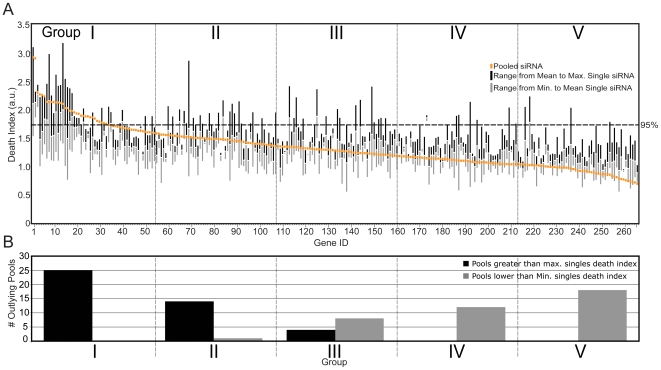
Comparison of single and pooled siRNA screens reveals the level of phenotypic penetrance for each siRNA. (A) A distribution of the death indices of single and pooled siRNAs. Genes are sorted from greatest to lowest siRNA pool death indices. The range of death indices attributed to the corresponding individual siRNAs are shown for each gene. (B) Graphic representation of non-overlapping phenotypes for pools and corresponding single siRNAs. Pooled siRNAs were sorted into five equally sized groups of decreasing death indices. The amount of pools with death indices higher than the maximal corresponding single siRNA are shown for each group as black columns. The amount of pools with death indices lower than the minimal corresponding single siRNA are shown for each group as grey columns.

The greater apparent frequency of hits in screens performed with siRNA pools prompted us to examine the severity of the phenotypes described for pooled and single siRNA duplexes. To this end, we arranged each gene product in descending order of death index from the pooled screen and plotted the range of death indices for the single siRNA duplexes that target the same gene product (Figure 4A). A cursory examination of the corresponding death indices reveals a broad range of death indices for a large number of single siRNAs that target a common gene. This is a common feature of individual siRNA molecules and likely reflects that fact that many siRNA duplexes fail to significantly deplete the target protein.

We were surprised to note that the death index attributed to siRNA pools often fell outside the range of death indices attributed to the corresponding set of individual siRNA duplexes. In total, we identified 82 of 265 cases (31%) where the death index of the siRNA pool did not overlap with the range of death indices for the corresponding individual siRNA duplexes. Close examination of the siRNA pools with death indices outside the corresponding single siRNA ranges revealed a particularly intriguing feature. We divided the siRNA pools into five equally sized groups (53 pools per group) that ranged from pools with the highest death index to pools with the lowest death index (Figure 4A). In the group of pools with the highest death indices, 25 had death indices that were outside the range of the corresponding single siRNA duplexes. Strikingly, the death index for each one of the siRNA pools was greater than the maximum death index of the individual siRNA duplexes (Figure 4B). We noticed an identical phenomenon when we examined the opposite end of the phenotypic spectrum. Of the 53 pooled siRNAs with the lowest death index, 18 had death indices that were outside the range of the corresponding single siRNA duplexes. In each case, the death index for the siRNA pool was lower than the lowest death index identified for the corresponding single siRNA duplexes. These data suggest that the phenotypic penetrance of siRNA pools often exceeds the phenotypic penetrance of any one of the corresponding single siRNA duplexes.

### A Comparison of Pooled and Single siRNA Screen Data

The frequent disparity between phenotypic penetrance for single duplexes and siRNA pools led us to question the phenotypic relationship between both methods of siRNA delivery. For these comparisons, we postulated that a phenotype from an siRNA pool may represent the mean phenotype of the individual siRNA duplexes; the median phenotype of the individual duplexes; or the optimal phenotype of the individual duplexes. To distinguish these possibilities, we plotted the death indices for the siRNA pools against the mean, median and optimal death indices from the corresponding single siRNA duplexes. We considered two possible definitions of “optimal” phenotype: one where the siRNA duplex with the most extreme death index constitutes the optimal death index (single optimal); and one where the mean of the two greatest death indices from the single siRNA duplexes constitute the optimal death index (cumulative optimal). The former definition favors a scenario where a very limited number of highly efficient on-target siRNAs determine the loss-of-function phenotype. The latter definition is more biased to a scenario where the loss-of-function phenotype represents the cumulative effect of all on-target siRNAs.

**Figure 5:**
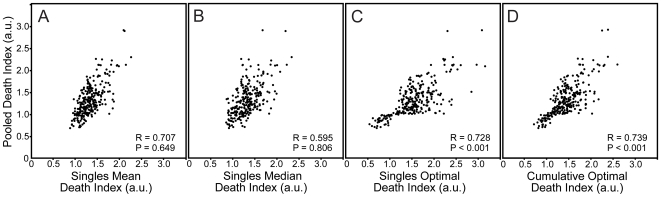
Comparative analysis of all pooled siRNA and single siRNA death indices. (A) The x-axis shows the mean death index of all three siRNAs tested per gene. (B) The x-axis shows the median death index of all three siRNAs tested per gene. (C) The x-axis shows the greatest single death index of all three siRNAs tested per gene. (D) The x-axis shows the mean of the two greatest death indices of all three siRNAs tested per gene. The correlation and statistical significance between the death index values of the mean single and pooled siRNA is indicated.

For each scenario, we determined the correlation coefficient and used paired students t-tests to determine the significance of overlap between the two death indices. In each case, we noticed a general overlap between the corresponding death indices (Figure 5A–D). These data indicate a broad phenotypic consensus between siRNA pools and multiple siRNA duplexes and argue that screens with siRNA pools are equally efficient at generating loss-of-function phenotypes as screens performed with multiple individual siRNAs. Strikingly, paired students t-tests revealed a significant phenotypic correlation between siRNA pools and the optimal death index (cumulative or single) for the corresponding single siRNA duplexes. Thus, it appears that siRNA pools often generate the maximal phenotype for the corresponding single duplexes.

## Discussion

RNAi-based loss-of-function studies open entirely new perspectives in cell biology, particularly for the identification of biomarkers or pharmacologically relevant targets in critical disease states. As with many genetic techniques, groundbreaking studies in the model invertebrates *C. elegans* and *Drosophila melanogaster* guided subsequent developments in mammalian systems. In *Drosophila* and *C. elegans*, RNAi involves the introduction of large dsRNA molecules typically greater than 250 bp into target cells. The large duplexes are diced internally into numerous small 21mer duplexes, which feed the classical RISC/RNAi pathway. Thus, RNAi experiments in invertebrates are, by design, performed with large pools of individual siRNA duplexes.

As large dsRNA molecules induce non-specific interferon responses in mammalian cell lines [13], alternative strategies are required for targeted knock-down studies. Typically, investigators use lipid-based or viral vehicles to deliver a small number (1 to 4) of non-overlapping siRNAs to target cells. These siRNA molecules are either delivered individually or as pools. Despite the clear experimental differences between the separate delivery of multiple individual duplexes and a pool of the same duplexes, there are very little data on the phenotypic consequences of such an approach. The bulk of recommendations on the relative advantages and disadvantages of pooled or single siRNAs are given by product vendors with vested commercial interests in the outcome. For example, a recent review of siRNA technologies specifically discussed the relative merits of pooled versus single siRNAs from the perspective of various commercial vendors of siRNA libraries [14]. Unsurprisingly, the opinions were immediately aligned with the product portfolio of the individual companies – the leading vendor of siRNA pools recommended pools and vendors of individual duplexes strongly cautioned against the use of pools. In this study, we set out to prepare an unbiased comparison of the phenotypes generated by siRNA pools and the parental single siRNAs in a simple functional assay. Given the tremendous potential of siRNA screens for the identification of biomarkers or pharmacological targets and the explosive growth in prominent studies based on high-throughput siRNA studies, we believe our findings are of considerable value to a large community of biological researchers.

Perhaps the most significant aspect of our findings is the fact that the phenotypic penetrance for siRNAs pools is frequently more severe than for any of the parental siRNA duplexes. Evaluation of our phenotypic data suggests that the phenotype associated with a pool of siRNAs most closely resembles the cumulative functional phenotype of the parental siRNA duplexes. The most parsimonious explanation for this observation is that the effective target knock-down achieved with an siRNA pool is a combination of the knock-downs achieved by the constituent duplexes. Thus, in a case where several individual duplexes yield partial knock-downs that are insufficient to uncover meaningful phenotypes, a combination of the individual knock-downs may translate into a more robust and detectable phenotype. Most siRNA design algorithms are based on empirical observations of the sequence composition of effective on-target siRNAs. Advances in siRNA design algorithms allow vendors to make general guarantees about the likelihood of transcript destruction upon the purchase of a set of non-overlapping siRNAs that target a specific transcript. However, it is not possible to predict the success of each individual siRNA, as the exact cellular context in which siRNAs interact with target transcripts remains unresolved. For example, siRNA design algorithms do not address critical issues such as secondary structures within specific target transcripts or accessibility issue presented by RNA protein complexes. siRNA pools may partially compensate for these shortcomings by combining several weak knock-down phenotypes to generate a more penetrant phenotype.

As siRNA pools often generate more penetrant phenotypes than any of the corresponding single duplexes, we also expect that screens with pools will identify a greater number of putative hits in the primary screen. Consistent with this hypothesis, we note that standard analysis of our siRNA pool data identified 33 putative hits (12% hit rate), while analysis of our single siRNA data identified 16 medium-confidence hits (6% hit rate) and only three high-confidence hits (1% hit rate). Based on our own work and data from other siRNA screens, we consider it highly probable that some of the putative hits from the siRNA pools are false positives. However, we believe that the effort required to identify false positives in secondary analysis is preferable to the loss represented by false negatives in misguided primary analysis.

In summary, our data argue that screens performed with siRNA pools identify a larger set of hits than screens performed with single siRNA duplexes and that the phenotypic penetration for siRNA pools is often greater that any of the individual siRNAs. Thus, we conclude that screens with siRNA pools represent an optimal approach in terms of phenotypic strength, throughput and reagent efficiency.

## Materials and Methods

### siRNA Library of Human Phosphatases

To obtain the targeted knockdown of 265 phosphatases we used the Ambion Silencer® human phosphatase-specific siRNA library (Applied Biosystems). The library is composed of three sequence-independent siRNAs per gene target and was handled according to manufacturer's protocol. To obtain the pooled siRNA library, equal volumes from all three target-redundant single siRNA libraries were combined. The single and pooled siRNA libraries were prepared to stock concentrations of 200 nM, thereby establishing the concentration of each unique siRNA in the pooled library at 66.67 nM.

### Single and Pooled siRNA Screens Using the Resazurin Viability Assay

For siRNA library screening in a 96 well plate format, all steps were performed on a sterile automated liquid handling platform (Janus® PerkinElmer). HeLa cells of an identical passage number at 8.67×10^4^ cells/ml were reverse transfected with siRNA at a final concentration of 20 nM using DharmaFect 1 transfection reagent (Dharmacon). After transfection, cells were incubated with siRNA under standard mammalian cell culture conditions for 72 h. Following the RNAi incubation period, cells were either treated with cell culture medium alone or with a mixture of Tumor Necrosis Factor alpha (Roche) and Cyclohexamide (Sigma) in culture medium to a final concentration of 20 ng/ml and 5µg/ml respectively. The redox dye, resazurin (Sigma), was added to cells 13.5 h after treatment and incubated with cells for 1.5 h before reading fluorescence signal on a multi-label plate reader (EnVision® PerkinElmer).

### Statistical and Graphical Analyses

To analyze the screen data, confidence intervals were established from the standard deviation of the single siRNA screens from the non-silencing control siRNA on the respective plates. To establish a simplified view of gene clusters with differential involvement in modulating TNF/CHX-induced death, cluster analysis was performed using the Cluster 3.0 program. Data from 265 normalized death indices measured from single and pooled screens were organized by hierarchical clustering. Euclidean distance between normalized death indices was used as the metric. The results of this cluster analysis are represented as a heatmap generated by Java TreeView 1.1.3.

## Supporting Information

Table S1 Normalized viability scores and death indices for each individual siRNA and all siRNA pools.
(0.27 MB XLS)
